# Morphometric grading of breast cancer: thresholds for tubular differentiation

**DOI:** 10.1054/bjoc.2000.1222

**Published:** 2000-04-27

**Authors:** P Kronqvist, T Kuopio, Y Collan

**Affiliations:** Department of Pathology, University of Turku, Kiinamyllynkatu 10, Turku, FIN-20520, Finland; Jyväskylä Central Hospital, Keskussairaalantie 19, Jyväskylä, FIN-40620, Finland

**Keywords:** breast carcinoma, prognosis, grading, tubular differentiation

## Abstract

We evaluated the degree of tubular differentiation in 172 samples of invasive ductal breast cancer in order to determine numerical thresholds for histological breast cancer grading. The tubular differentiation in each sample was defined as the fraction of fields showing tubular differentiation (FTD). The analysis was based on Kaplan–Meier curves reflecting survival and recurrence of disease, univariate and multivariate analyses of Cox's regression, and maximum efficiencies of ROC analysis. The minimum *P* -value cut-off for FTD was determined at 59%. The practical interpretation is that tubular differentiation in the neoplasm observed in at least 60% of microscopical fields in the tumour area indicates favourable prognosis of disease. The relative risks for breast cancer death for patients with FTD below 59% as compared with those with FTD above 59% were 6.7- and 6.3-fold (univariate and multivariate analyses respectively). Another threshold could be determined at FTD 23%, although this threshold was associated with clearly lower statistical significancies. The paper introduces two possible solutions for application of the thresholds to the morphometric breast cancer grading system. The study also emphasizes the clinical relevance of the evaluation of tubular differentiation in breast cancer. The consistent morphometric evaluation method was vital in allowing the full weight of the biological significance of tubular differentiation to emerge. © 2000 Cancer Research Campaign


*British Journal of Cancer* (2000) **82**(10), 1656–1661
© 2000 Cancer Research Campaign
DOI: 10.1054/ bjoc.2000.1222, available online at http://www.idealibrary.com on IDEAL®

### Morphometric grading of breast cancer: thresholds for tubular differentiation

**P Kronqvist[1], T Kuopio[2] and Y Collan[1]**

[1]Department of Pathology, University of Turku, Kiinamyllynkatu 10, FIN-20520 Turku, Finland; [2]Jyväskylä Central Hospital, Keskussairaalantie 19, FIN-40620 Jyväskylä, Finland

**Summary** We evaluated the degree of tubular differentiation in 172 samples of invasive ductal breast cancer in order to determine numerical thresholds for histological breast cancer grading. The tubular differentiation in each sample was defined as the fraction of fields showing tubular differentiation (FTD). The analysis was based on Kaplan–Meier curves reflecting survival and recurrence of disease, univariate and multivariate analyses of Cox's regression, and maximum efficiencies of ROC analysis. The minimum *P*-value cut-off for FTD was determined at 59%. The practical interpretation is that tubular differentiation in the neoplasm observed in at least 60% of microscopical fields in the tumour area indicates favourable prognosis of disease. The relative risks for breast cancer death for patients with FTD below 59% as compared with those with FTD above 59% were 6.7- and 6.3-fold (univariate and multivariate analyses respectively). Another threshold could be determined at FTD 23%, although this threshold was associated with clearly lower statistical significancies. The paper introduces two possible solutions for application of the thresholds to the morphometric breast cancer grading system. The study also emphasizes the clinical relevance of the evaluation of tubular differentiation in breast cancer. The consistent morphometric evaluation method was vital in allowing the full weight of the biological significance of tubular differentiation to emerge. © 2000 Cancer Research Campaign

**Keywords**: breast carcinoma; prognosis; grading; tubular differentiation

Substantial evidence in medical literature indicates a relationship between the prognosis of invasive ductal breast cancer and the degree of histological differentiation (Roberts and Hahnel, 1981; Tosi et al, 1986; Clayton, 1991; Lipponen et al, 1991; Fisher et al, 1993; Garne et al, 1994). Evaluation of the degree of tubular differentiation in the tumour tissue is part of histological malignancy grading of invasive breast cancer (Patey and Scarff, 1928; Bloom and Richardson, 1957; WHO, 1981). The assessment of tubular differentiation in breast cancer grading has, however, often been accused of inaccuracy and poor reproducibility. Therefore the contribution of the assessment of tubular differentiation to breast cancer prognostication and treatment decisions may be underestimated.

Previously, we have introduced numerical thresholds for nuclear grade and mitotic activity in breast cancer based on follow-up information (Kronqvist et al, 1998*a*, 1998*b*). The thresholds were part of the development of the morphometric grading system which we are designing for invasive ductal breast cancer. We have now set out to determine corresponding thresholds for tubular differentiation. The final aim is to augment the prognostic potential and improve the reproducibility of breast cancer grading with the help of quantitative histological methods.

*Received 27 August 1999*
*Revised 6 January 2000*
*Accepted 17 January 2000*

*Correspondence to:* P Kronqvist

### Materials and methods

### Patients

The study comprises 172 cases of invasive ductal breast cancer diagnosed and treated at Turku University Hospital in the years 1989–1991 (Table 1). Complete follow-up histories and perioperative specimens from the primary tumours were available for all patients. The patients with previously detected breast cancer in unilateral or contralateral breast were excluded from the material. Moreover, we left out of the analysis all cases of M1 stage if the distant metastasis was detected within 1 month of diagnosis. Metastases were detected by routine chest and bone radiographs, laboratory test reflecting bone and liver metabolism and by cytological and histological samples when obtainable. All patients were treated by radical or modified radical mastectomy with axillary evacuation. None of the patients received preoperative radiation therapy or other preoperative adjuvant treatments. Two different post-operative adjuvant treatment protocols were applied in our hospital during the follow-up period of our breast cancer material. At the end of the 1980s, anti-oestrogen (post-menopausal patients) and cytostatic (premenopausal) therapy was given to all patients with T4 stage disease. Patients with histologically verified metastasis in four or more axillary lymph nodes or in one apical lymph node received the same treatment. At the beginning of the 1990s, anti-oestrogen treatment or cytostatic drugs were given to all patients with histologically verified axillary lymph node metastases. In our material post-operative early adjuvant systemic therapy was given to 50 patients, 36 of whom received endocrine therapy and 14 chemotherapy. The causes of death were collected from autopsy reports, death certificates and patient files. The overall survival rate was 67.4% and breast cancer-related survival rate was 73.4% as determined at 5 years of follow-up by excluding patients dead of causes other than breast carcinoma.

**Table 1 Characteristics of the patients (*n* = 172)**

| | |
|---|---|
| Mean age at diagnosis (range) | 59.1 years (32.6–86.5 years) |
| Menopausal status | |
| No. of premenopausal women | 56 (32.6%) |
| No. of post-menopausal women | 116 (67.4%) |
| Axillary lymph node status | |
| No. of positive patients | 72 (41.9%) |
| No. of negative patients | 100 (58.1%) |
| Mean tumour size (range) | 2.9 cm (0.6–15.0 cm) |
| Mean follow-up time (range) | 5 years 8 months (5 months – 8 years 11 months) |
| No. of cases with recurrence | 66 (38.4%) |
| Causes of death during follow-up | |
| Breast cancer | 42 (24.4%) |
| Other cancer | 5 (2.9%) |
| Other | 9 (5.2%) |

### Evaluation of tubular differentiation

The histological samples used in assessments of tubular differentiation were fixed in buffered formalin (pH 7.0), embedded in paraffin, sectioned at 5 μm and stained with haematoxylin and eosin.

To begin with the tubular measurements we chose the most representative slide of each case, placing special emphasis on the quality of the histological details. Tubular differentiation was evaluated in each sample as the fraction of fields showing tubular differentiation (FTD) (Kronqvist et al, 1999). According to this method tubular differentiation was assessed in the whole tumour area. The sample was screened field by field with ×25 magnification (field diameter 710 μm) and the presence or absence of malignant tubular structures in each microscopic field was registered. By this method the field was registered positive if a single undoubtable malignant tubular structure was identified. The final result was the fraction of fields presenting tubular differentiation. This assessment method has been developed in our research group and is especially recommended because it has turned out to be the most efficient and fastest way to evaluate in quantitative terms the tubular differentiation in invasive breast cancer. In a previous paper comparing several evaluation methods for tubular differentiation (Kronqvist et al, 1999), FTD showed out to be the most practical, accurate and reproducible way to determine tubular differentiation in invasive ductal breast cancer.

In the evaluations, special emphasis was placed on histological identification of the malignant tubuli. The main criteria for registering a tubulus was a definite lumen within a tubular or alveolar structure created by surrounding malignant epithelial cells. Special consideration was taken not to mistake adipocytes, central necrosis or clefts due to shrinkage artifacts as tubular spaces. Luminal structures in cribriform malignant epithelium were not counted either.

### Statistical analysis

The results were analysed with the SAS statistical package (SAS® System for Windows™ release 6.12, SAS Institute Inc., Cary, NC, USA) in the whole patient material, and in subgroups of samples divided by the patients' age and axillary lymph node status at diagnosis and tumour size. The prognostic value of all possible cutpoints for FTD was tested throughout their range to find the optimal threshold for tubular differentiation. The prognostic value of the tested cut-offs was estimated on the basis of outcome in patient groups with FTD below and above the cut-off. For this purpose Kaplan–Meier curves (Cutler and Ederer, 1958) were drawn for each cut-off based on survival of disease and disease-free period, and the curves were tested for statistical significance with the help of log-rank test (*P*-values and $\chi^2$ values). The $\chi^2$ of log-rank tests were summarized in diagrams showing the variation of statistical significance associated with each tested cut-off. The cut-off resulting in the clearest rise in statistical significance was considered to best stratify the cases with different prognosis of disease and represent the most reliable thresholds for classification of patients on the basis of FTD. The false-positive rate of the minimum *P*-value approach was taken into account and the corrected *P*-value ($P_{cor}$) calculated as suggested by Altman and co-workers ($\varepsilon = 0.1$) (Altman et al, 1994). Univariate and multivariate analyses based on Cox's regression were applied to evaluate the prognostic significance of tubular differentiation. Associations between different prognostic factors and breast cancer outcome were quantified with ratios indicating relative risk (RR) of breast cancer recurrence or death and the corresponding 95% confidence intervals (95% CI).

The threshold and the confidence associated with the classification by the threshold was determined with the help of grading efficiencies (GE) (Galen and Gambino, 1975; Collan, 1989; Collan et al, 1992) and Receiver Operating Characteristic (ROC) curves (Hanley and McNeil, 1982; Beck and Schultz, 1986; Zweig and Campbell, 1993; Kairisto and Poola, 1995). The efficiencies and the ROC curves were produced with the help of the GraphROC software (GraphROC for Windows, University of Turku, Department of Clinical Chemistry, Turku, Finland) (Kairisto and Poola, 1995) and they represent the potential of the method to distinguish live patients from those dead from breast cancer at 5 years of follow-up.

### RESULTS

The mean fraction of FTD in our material was 30.0% (median 22.2%, standard deviation 28.2%). When applying the established thresholds of subjective tubular grading (10% and 75% of tumour area showing tubule formation)[22,23] to our results of FTD, 9.3% of the cases indicated favourable, 54.7% intermediate and 36.0% unfavourable prognosis of disease.

Figure 1 demonstrates the distribution of chi-values of log-rank tests associated with all the possible cut-offs of FTD determined at 1% intervals. The peak in statistical significance ($P = 0.0026$, $P_{cor} = 0.0215$) at FTD 59% was considered to represent the most reliable threshold ($FTD_{59\%}$) for classifying patients according to tubular differentiation. Moreover, we observed a smaller but still statistically significant peak at FTD 23% (threshold $FTD_{23\%}$). Figure 2 demonstrates the potential of the determined thresholds to stratify between patients with favourable, intermediate and unfavourable outcome of disease. The thresholds $FTD_{59\%}$ and $FTD_{23\%}$ were identical in analyses based on breast cancer survival and on disease-free period in the whole material. Both thresholds could be detected also in analyses of post-menopausal patients (Table 2). In most prognostic subgroups, however, only $FTD_{59\%}$ could be verified as a threshold. $FTD_{23\%}$, in turn, was detected as the only threshold among axillary lymph node-positive patients. No statistically significant threshold could be found among cases of small tumour size (equal to or below 2 cm in diameter).

*© 2000 Cancer Research Campaign*

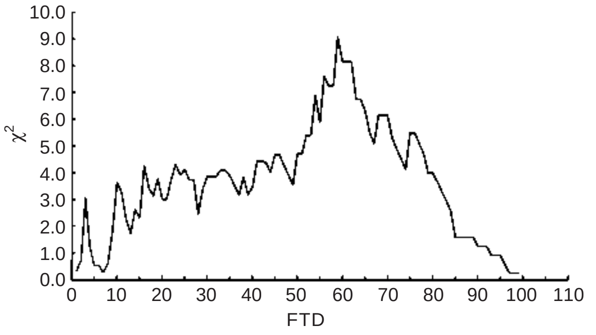

**Figure 1** The distribution of $X^2$ of log-rank tests associated with the cut-offs for the fraction of tubular differentiation (FTD) in 172 cases of invasive breast cancer. The cut-off with the clearest rise in statistical significance at FTD 59% was considered to represent the most reliable thresholds for classification of patients on the basis of survival and recurrence of disease. Another peak showing lower but still statistically significant chi-square values was found at FTD 23%

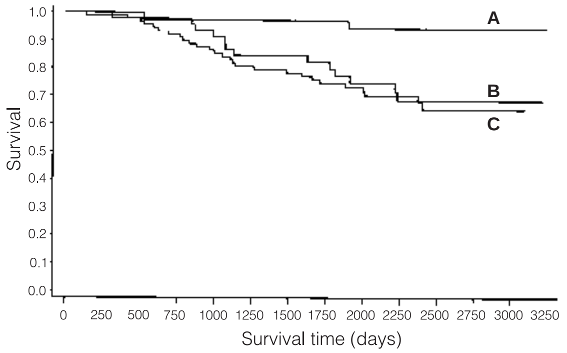

**Figure 2** The determined threshold, $FTD_{59\%}$, for tubular differentiation clearly stratify the patients with favourable (A) and intermediate (B) prognosis ($P = 0.009$). The threshold $FTD_{23\%}$ could be applied to distinguish those patients with the worst outcome of disease (C) ($P = 0.041$)

**Table 2** Thresholds for FTD determined on the basis of follow-up information on breast cancer survival of 172 breast cancer patients

| Group of patients | Lower threshold | Higher threshold |
|---|---|---|
| ALL | 23% | 59% |
| Premenopausal | | 59% |
| Postmenopausal | 23% | 59% |
| Tumour diameter < 2 cm | | |
| Tumour diameter ≥ 2 cm | | 59% |
| Node – | | 59% |
| Node + | 23% | |

All = all patients. Node – = axillary lymph node-negative patients. Node + = axillary lymph node-positive patients.

The table includes results of analyses in the whole material and in prognostic subgroups stratified according to menopausal and axillary lymph node status at diagnosis, and tumour size. The thresholds shown divide the patient material into two groups the survival of which is different at a significance level of $P < 0.05$. When several significant cutpoints were found the threshold showing the lowest $P$-value was chosen.

Table 3 summarizes the relative risks (RRs) of univariate analyses describing the risk of breast cancer death associated with feature values below the thresholds as compared with feature values above the thresholds. In results of the whole material, $FTD_{59\%}$ was the most powerful predictor of survival with a 6.7-fold risk of breast cancer death. $FTD_{23\%}$ was associated with a 1.9-fold risk of breast cancer death. Among axillary lymph node-positive patients, however, $FTD_{23\%}$ was the strongest significant prognosticator for survival.

In multivariate analysis of the whole material among $FTD_{59\%}$, menopausal status and axillary lymph node status at diagnosis (Table 4), $FTD_{59\%}$ was associated with a 6.3-fold risk of breast cancer death. Axillary lymph node status was verified as an independent prognostic factor in the whole material, among post-menopausal patients and patients with small tumour size (tumour diameter equal to or below 2 cm).

The grading efficiencies of $FTD_{59\%}$ and $FTD_{23\%}$ at 5 years follow-up were determined with the help of ROC-analysis. The GEs of the cut-offs of subjective evaluation of tubular differentiation in the SBR classification (Ellis and Elston, 1991; Simpson and Page, 1994) (0.588 and 0.563 for the cut-offs of 10% and 75% respectively) were inferior to the FTD-based morphometric thresholds (0.620 and 0.591 for $FTD_{59\%}$ and $FTD_{23\%}$ respectively). The area under curve (AUC) of ROC analysis in the whole material is 0.611, indicating moderate classification potential of the method. The maximum efficiency point of ROC analysis in the whole material at 58.4% supports the conclusion of $FTD_{59\%}$ as the optimal threshold for tubular grading. Among node-positive cases the maximum efficiency point at 22.2% corresponds to the threshold $FTD_{23\%}$.

### DISCUSSION

Based on follow-up information of breast cancer survival and recurrence the optimal (minimum $P$-value) threshold for tubular differentiation could be determined at TDF 59%. The practical interpretation is that tubular differentiation observed in at least 60% of the microscopical fields in the tumour area indicates favourable prognosis of disease. $FTD_{59\%}$ is relevant also for prognostication in pre- and post-menopausal and axillary lymph node negative subgroups of patients as well as in cases of large tumour size (diameter above 2 cm). Another threshold detected at FTD 23% was efficient especially in predicting the prognosis of axillary lymph node-positive patients. This threshold could be applied in identifying those patients with the worst outcome of disease. In medical literature, the 'minimum $P$-value approach' has been considered contradictory (Altman et al, 1994) which suggests that the results of this type of statistical analysis should be interpreted cautiously. In the present study, however, the reliability of the results is emphasized by the fact that the same numerical thresholds for tubular differentiation could also be found in univariate and multivariate analysis of Cox's regression, and in ROC-analysis based on the follow-up information of the patient material. The relative risks for breast cancer death associated with $FTD_{59\%}$ were 6.7- and 6.3-fold in univariate and multivariate analyses respectively. Concluding from Kaplan–Meier curves of breast cancer survival the determined thresholds for tubular differentiation very efficiently stratified the patients with different outcome of disease, especially in favourable and intermediate prognostic groups (threshold $FTD_{59\%}$).

In light of previous medical literature the prognostic value of tubular differentiation in invasive breast cancer is conflicting. Many papers report that tubular differentiation is a noteworthy

 © 2000 Cancer Research Campaign

**Table 3** Univariate analyses performed in the whole material of 172 patients on the determined thresholds for fraction of tubular differentiation, $FTD_{59\%}$ and $FTD_{23\%}$, and the patients' menopausal status, axillary lymph node status and tumour size

| Group of patients | Feature | *P* | RR | 95% CI |
|---|---|---|---|---|
| ALL | **$FTD_{59\%}$** | **0.009** | **6.7** | **1.7–27.8** |
| | **Nodal status** | **0.002** | **2.7** | **1.5–5.1** |
| | **$FTD_{23\%}$** | **0.041** | **1.9** | **1.0–3.6** |
| | Tumour size | 0.164 | 1.5 | 0.8–2.9 |
| | Menopausal status | 0.470 | 0.8 | 0.4–1.5 |
| Premenopausal | *$FTD_{59\%}$* | **0.072** | **6.4** | **0.8–48.4** |
| | *Nodal status* | *0.055* | *2.6* | *1.0–7.1* |
| | Tumour size | 0.254 | 1.9 | 0.6–5.3 |
| | $FTD_{23\%}$ | 0.992 | 1.0 | 0.4–2.7 |
| Post-menopausal | **$FTD_{23\%}$** | **0.011** | **3.3** | **1.3–8.2** |
| | **Nodal status** | **0.010** | **2.9** | **1.3–6.5** |
| | *$FTD_{59\%}$* | **0.054** | **7.1** | **1.0–52.6** |
| | Tumour size | 0.417 | 1.4 | 0.6–3.0 |
| Tumour diameter ≤ 2 cm | **Nodal status** | **0.021** | **3.0** | **1.2–7.4** |
| | $FTD_{23\%}$ | 0.182 | 1.7 | 0.3–4.0 |
| | Menopausal status | 0.447 | 0.7 | 0.3–1.6 |
| | $FTD_{59\%}$ | 0.991 | NS | NS |
| Tumour diameter > 2 cm | $FTD_{59\%}$ | 0.172 | 2.8 | 0.6–12.3 |
| | $FTD_{23\%}$ | 0.132 | 2.2 | 0.8–5.9 |
| | Nodal status | 0.105 | 2.2 | 0.8–5.8 |
| | Menopausal status | 0.994 | 1.0 | 0.4–2.8 |
| Node – | $FTD_{23\%}$ | 0.765 | 1.2 | 0.4–3.1 |
| | Tumour size | 0.977 | 1.0 | 0.4–2.7 |
| | Menopausal status | 0.488 | 0.7 | 0.3–1.9 |
| | $FTD_{59\%}$ | 0.992 | NS | NS |
| Node + | **$FTD_{23\%}$** | **0.035** | **2.6** | **1.1–6.1** |
| | $FTD_{59\%}$ | 0.115 | 3.2 | 0.8–13.5 |
| | Tumour size | 0.529 | 1.3 | 0.6–3.1 |
| | Menopausal status | 0.564 | 0.8 | 0.4–1.8 |

ALL = all patients. Node – = axillary lymph node-negative patients. Node + = axillary lymph node-positive patients. In addition to the P values, risk ratios (RR) of breast cancer death with 95% confidence intervals (95% CI) in the whole material and in the prognostic subgroups are shown. RRs are presented in size order and the level of statistical significance is indicated ($P < 0.05$ in bold, $0.1 < P < 0.05$ in italics).

**Table 4** Multivariate analyses performed in the whole material of 172 patients on the determined thresholds for fraction of tubular differentiation, $FTD_{59\%}$ and $FTD_{23\%}$, and the patients' menopausal status, and axillary lymph node status.

| Group of patients | Feature | *P* | RR | 95% CI |
|---|---|---|---|---|
| ALL[a] | **$FTD_{59\%}$** | **0.011** | **6.3** | **1.5–26.2** |
| | **Nodal status** | **0.003** | **2.6** | **1.4–4.8** |
| | Menopausal status | 0.285 | 0.7 | 0.4–1.3 |
| | **Nodal status** | **0.002** | **2.7** | **1.4–5.0** |
| | *$FTD_{23\%}$* | **0.051** | **1.9** | **1.0–3.5** |
| | Menopausal status | 0.296 | 0.7 | 0.4–1.3 |
| | <u>Feature with the highest RR</u> | | | |
| | Analysis including $FTD_{59\%}$ | | | |
| Premenopausal[a] | $Tub_{59\%}$ | 0.101 | 5.5 | 0.7–42.1 |
| Post-menopausal[a] | *$Tub_{59\%}$* | **0.052** | **7.3** | **1.0–53.7** |
| Tumour diameter ≤ 2 cm[b] | **Nodal status** | **0.015** | **3.2** | **1.3–8.0** |
| Tumour diameter > 2 cm[b] | $Tub_{59\%}$ | 0.211 | 2.6 | 0.6–11.8 |
| Node –[c] | Menopausal status | 0.263 | 0.6 | 0.2–1.5 |
| Node +[c] | $Tub_{59\%}$ | 0.119 | 3.2 | 0.7–13.4 |
| | <u>Analysis including $FTD_{23\%}$</u> | | | |
| Premenopausal[a] | *Nodal status* | *0.055* | *2.6* | *1.0–7.1* |
| Post-menopausal[a] | **$FTD_{23\%}$** | **0.013** | **3.2** | **1.3–8.0** |
| Tumour diameter ≤ 2 cm[b] | **Nodal status** | **0.020** | **3.0** | **1.2–7.6** |
| Tumour diameter > 2 cm[b] | *Nodal status* | *0.087* | *2.3* | *0.9–6.1* |
| Node –[c] | $FTD_{23\%}$ | 0.725 | 1.2 | 0.4–3.2 |
| Node +[c] | **$FTD_{23\%}$** | **0.033** | **2.6** | **1.1–6.1** |

ALL = all patients, Node− = axillary lymph node-negative patients, Node+ = axillary lymph node-positive patients. [a]Adjusted for axillary lymph node status and tumour size. [b]Adjusted for axillary lymph node status. [c]Adjusted for tumour size. In addition to the P values, risk ratios (RR) of breast cancer death with 95% confidence intervals (95% CI) in the whole material and in the prognostic subgroups are shown. RRs are presented in size order and the level of statistical significance is indicated ($P<0.05$ in bold, $0.1<P<0.05$ in italics).

© 2000 Cancer Research Campaign

**Table 5** Comparison of the traditional and introduced scores for histological grading of invasive ductal breast cancer

| Traditional | Introduced | Grade |
|---|---|---|
| 9 | 8 | III |
| 8 | 7 | |
| 7 | 6 | II |
| 6 | 5 | |
| 5 | 4 | I |
| 4 | 3 | |
| 3 | | |

prognosticator of breast cancer outcome (Fisher et al, 1975, 1984; Parl and Dupont, 1982; Davis et al, 1986; Fisher, 1986; Theissig et al, 1990; Dalton et al, 1994; Robbins et al, 1995). On the other hand, there is an abundance of papers stating that tubular differentiation lacks prognostic significancy and is inferior to the other two features of histological breast cancer grading (Black et al, 1955; Baak et al, 1985; Rank et al, 1987; Le Doussal et al, 1989; van der Linden et al, 1989; Theissig et al, 1990; Clayton, 1991; Lipponen et al, 1991; Parham et al, 1992; Schumacher et al, 1993; Dalton et al, 1994). In our opinion, these contradictions reflect the subjectivity of the evaluation methods rather than the lack of biological significance of tubular differentiation. Neither the traditional (Patey and Scarff, 1928; Bloom and Richardson, 1957) nor the modified grading systems of breast cancer (WHO, 1981; Haybittle et al, 1982; Todd et al, 1987; Ellis and Elston, 1991; Simpson and Page, 1994) give detailed guidelines for identification of tubular structures or numerical quantification of the degree of tubular differentiation in the tumour tissue. In the present study tubular differentiation was registered as fraction of fields with tubular differentiation (FTD) (Kronqvist et al, 2000). The method has been developed in our research group and proven accurate, reliable and practical. One advantage of the method is that the assessment is performed field by field which directly results in a numerical estimate of tubular differentiation in the tumour area. Because in each field only the presence or absence of clearly defined tubuli is registered, the method is unambiguous, simple and relatively fast so that even a large section can be screened in fewer than 10 min. The obvious disadvantage of our method is, however, that the FTD is not directly comparable with the traditional subjective assessment method.

Our final aim is to adapt the results of the threshold analyses of tubular differentiation to the histological grading of invasive ductal breast cancer (Patey and Scarff, 1928; Bloom and Richardson, 1957; WHO, 1981). In order to maintain the traditional three-score system two alternative policies can, in our viewpoint, be followed. For the first, the two most efficient thresholds for tubular differentiation, $FTD_{59\%}$ and $FTD_{23\%}$, can be applied in the grading system, although the prognostic contribution of $FTD_{23\%}$ can be expected to be weak in many patient groups. Secondly, the scoring of the breast cancer grading system can be reorganized to apply only one threshold for tubular differentiation at FTD 59% (Table 5). This would result in a grading system with a total of 8 points which would equalize the score groups and simplify the allocation of the scores into their respective grades.

In light of our results tubular differentiation is an independent prognostic factor of invasive breast cancer. It is commonly believed that the prognostic value of histologically assessed tubular differentiation is presumably inferior to those of nuclear grade and mitotic activity. Future studies will show what are the prognostic contributions of the assessment of tubular differentiation in morphometric grading of invasive breast cancer (Kronqvist et al, 1998*a*, 1998*b*). The present study supports the application of the traditional three-subfeature grading system of invasive ductal breast cancer. Previous experiences together with our results suggest that tubular differentiation as evaluated by FTD could also be applicable in future automatic grading systems (Dufer et al, 1993).

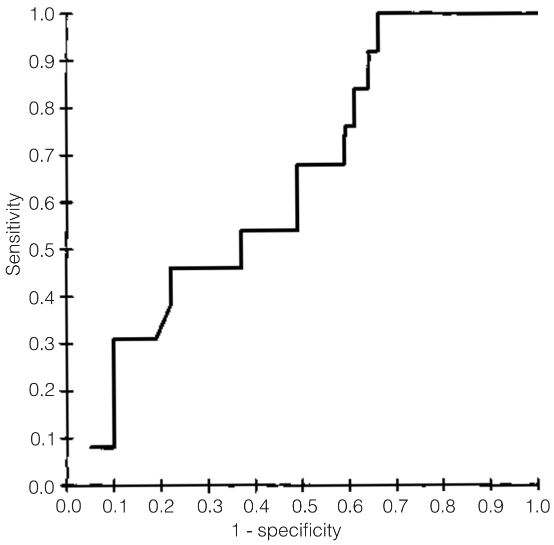

**Figure 3** ROC curve demonstrating the grading potential of FTD in the group of premenopausal patients. The area under the curve is 0.646

### ACKNOWLEDGEMENTS

This study was supported by grants from EVO funding from Turku University Hospital, Turku University Foundation, Women's Research Foundation, and Finnish Cancer Society.

### REFERENCES

Altman DG, Lausen B, Sauerbrei W and Schumecher M (1994) Dangers of using 'optimal' cutpoints in the evaluation of prognostic factors. *J Natl Cancer Inst* **86**: 829–835

Baak JPA, Van Dop H, Kurver PHJ and Hermans J (1985) The value of morphometry to classic prognosticators in breast cancer. *Cancer* **56**: 374–382

Beck JR and Schulz EK (1986) The use of relative operating characteristics (ROC) curves in test performance evaluation. *Arch Pathol Lab Med* **110**: 13–20

Berger U, Wilson P, McClelland KA, Davidson J and Coombes RC (1987) Correlation of immunocytochemically demonstrated estrogen receptor distribution and histopathologic features in primary breast cancer. *Hum Path* **18**: 1263–1267

Black MM, Opler SR and Speer FD (1955) Survival in breast cancer cases in relation to the structure of the primary tumour and regional lymph nodes. *Surg Gynecol Obstet* **100**: 543–552

Black MM, Barclay THC and Hankey BF (1975) Prognosis in breast cancer utilizing histological characteristics of the primary tumour. *Cancer* **36**: 2048–2055

Bloom HJG and Richardson WW (1957) Histological grading and prognosis in breast cancer. *Br J Cancer* **11**: 359–377

Clayton F (1991) Pathologic correlates of survival in 378 lymph node-negative infiltrating ductal breast carcinomas. Mitotic count is the best single predictor. *Cancer* **68**: 1309–1317

*© 2000 Cancer Research Campaign*

Collan Y (1989) General principles of grading lesions in diagnostic histopathology. *Pathol Res Pract* **185**: 539–543
Collan Y, Kuopio T and Alanen K (1992) Scope and concepts of quantitative histopathology. *Acta Stereol* **11**: 3–23
Cutler SJ and Ederer F (1958) Maximum utilization of the life table method in analyzing survival. *J Chron Dis* **8**: 699–712
Dalton LW, Page DL and Dupont WD (1994) Histologic grading of breast carcinoma. A reproducibility study. *Cancer* **73**: 2765–2770
Davis BW, Gelber D, Goldhirsh A, Hartmann WH, Locher GW, Reed R, Golouh R, Säve-Söderbergh J, Holloway L and Russel I (1986) Prognostic significance of tumour grade in clinical trials of adjuvant therapy for breast cancer with axillary lymph nodes metastasis. *Cancer* **58**: 2662–2670
Dufer J, Liataud-Roger F, Barbarin D and Coninx P (1993) Nucleus image analysis as a possible prognostic tool in grading breast cancer. *Biomed Pharmacother* **47**: 131–135
Ellis CW and Elston IO (1991) Pathological prognostic factors in breast cancer. I. The value of histological grade in breast cancer: experience from a large study with long-term follow-up. *Histopathology* **19**: 403–410
Fisher ER (1986) Prognostic and therapeutic significance of pathological features of breast cancer. *NCI Monogr* **1**: 29–34
Fisher ER, Gregorio RM and Fisher B (1975) The pathology of invasive breast cancer. A syllabus derived from findings of the National Surgical Adjuvant Breast Project (Protocol No. 4). *Cancer* **36**: 1–85
Fisher ER, Redmond C and Fisher B (1980) Histologic grading of breast cancer. *Path Annu* **15**: 239–251
Fisher ER, Sass R and Fisher B (1984) Pathologic findings from the national surgical adjuvant project for breast cancer (protocol no 4). Discriminants for tenth year treatment failure. *Cancer* **53**: 712–723
Fisher ER, Anderson S, Redmond C and Fisher B (1993) Pathologic findings from the National Surgical Adjuvant Breast Project Protocol B-06. 10 year pathological and clinical prognostic discriminants. *Cancer* **71**: 2507–2514
Galen RS and Gambino SR (1975) Beyond normality. In: *The Predicted Value and Efficiency of Medical Diagnosis*, pp. 49–51, 115–116. John Wiley and Sons: New York
Garne JP, Aspegren K, Linell F, Rank F and Ranstam J (1994) Primary diagnostic factors in invasive breast cancer with special reference to ductal carcinoma and histological malignancy grade. *Cancer* **73**: 1438–1448
Hanley JA and McNeil BJ (1982) The meaning and use of the area under a receiver operating characteristic (ROC) curve. *Radiology* **143**: 29–36
Haybittle JL, Blamey RW, Elston CW, Johnson J, Doyle PJ, Campbell FC, Nicholson RI and Griffiths K (1982) A prognostic index in primary breast cancer. *Br J Cancer* **45**: 361–366
Kairisto V and Poola A (1995) Software for illustrative presentation of basic clinical characteristics of laboratory tests – GraphROC for Windows. *Scand J Clin Lab Invest* **55**: 43–60
Kronqvist P, Kuopio T and Collan Y (1998*a*). Breast cancer prognostication: morphometric thresholds for nuclear grading. *Br J Cancer* **78**: 800–805
Kronqvist P, Kuopio T and Collan Y (1998*b*) Morphometric grading in breast cancer: thresholds for mitotic counts. *Hum Pathol* **29**: 1462–1468
Kronqvist P, Kuopio T, Pirvu C and Collan Y (1999) The fraction of fields showing neoplastic tubuli: practical estimate of tubular differentiation in breast cancer. *Histopathology* **35**: 401–410
Le Doussal V, Tubiana-Hulin M, Friedman S, Hacene K, Spyratos F and Brunet M (1989) Prognostic value of histologic grade nuclear components of Scarff–Bloom–Richardson (SBR). An improved score modification based on a multivariate analysis of 1262 invasive ductal breast carcinomas. *Cancer* **64**: 1914–1921
Lipponen PK, Collan Y and Eskelinen MJ (1991) Volume corrected mitotic index (M/V index), mitotic activity index (MAI), and histological grading in breast cancer. *Int Surg* **76**: 245–249
Parham DM, Hagen N and Brown RA (1992) Simplified method of grading primary carcinomas of the breast. *J Clin Pathol* **45**: 517–520
Parl FF and Dupont WD (1982) A retrospective cohort study of histologic risk factors in breast cancer patients. *Cancer* **50**: 2410–2416
Patey DH and Scarff RW (1928) The position of histology in the prognosis of carcinoma of the breast. *Lancet* **1**: 801–804
Rank F, Dombernowsky P, Bang-Jespersen NC, Pedersen BV and Keiding N (1987) Histologic malignancy grading of invasive breast carcinoma: A regression analysis of prognostic factors in low-risk carcinomas from a multicenter trial. *Cancer* **60**: 1299–1305
Robbins P, Pinder S, De Klerk N, Dawkins H, Harvey J, Sterett G, Ellis I and Elston C (1995) Histological grading of breast carcinomas: A study of interobserver agreement. *Hum Pathol* **26**: 873–879
Roberts AN and Hahnel R (1981) Oestrogen receptor assay and morphology of breast cancer. *Pathology* **13**: 317–325
Schumacher M, Schmoor C, Sauerbrei W, Schauer, A, Ummenhof L, Gatzmeier W and Rauschecker H (1993) The prognostic effect of histological tumour grade in node-negative breast cancer patients. *Breast Cancer Res Treat* **25**: 235–245
Simpson JF and Page DL (1994) Status of breast cancer prognostication based on histopathologic data. *Am J Clin Pathol* **102(suppl 1)**: S3–S8
Theissig F, Kunze KD, Haroske G and Meyer W (1990) Histological grading of breast cancer. Interobserver reproducibility and prognostic significance. *Path Res Pract* **186**: 732–736
Todd JH, Dowle C, Williams MR, Elston CW, Ellis IO, Hinton C, Blamey RW and Haybittle JL (1987) Confirmation of a prognostic index in primary breast cancer. *Br J Cancer* **56**: 489–492
Tosi P, Luzi P, Sforza V, Sanpietro R, Bindi M, Tucci E, Barbini P and Baak JP (1986) Correlation between morphometrical parameters and disease-free survival in ductal breast cancer treated only by surgery. *App Pathol* **4**: 33–42
Van Der Linden JG, Lindeman J, Baak JPA, Meijer CJLM and Hermans CJ (1989) The multivariate prognostic index and nuclear DNA content are independent prognostic factors in primary breast cancer patients. *Cytometry* **10**: 56–61
WHO (1981) Histological typing of breast tumours. In: *International Histological Classification of Tumours*; 2nd edn. World Health Organization: Geneva.
Zweig MH and Campbell G (1993) Receiver-operating characteristic (ROC) plots: a fundamental evaluation tool in clinical medicine. *Clin Chem* **39**: 561–577

© 2000 Cancer Research Campaign
